# Phytoplankton Biogeography and Community Stability in the Ocean

**DOI:** 10.1371/journal.pone.0010037

**Published:** 2010-04-02

**Authors:** Pedro Cermeño, Colomban de Vargas, Fátima Abrantes, Paul G. Falkowski

**Affiliations:** 1 Environmental Biophysics and Molecular Ecology Program, Institute of Marine and Coastal Sciences, Rutgers University, New Brunswick, New Jersey, United States of America; 2 Station Biologique de Roscoff, Evolution du Plancton et PaléoOcéans, Centre National de la Recherche Scientifique (CNRS) et Université Pierre et Marie Curie (UPMC), Roscoff, France; 3 Marine Geology Department, National Laboratory of Energy and Geology (LNEG), Alfragide, Portugal; 4 Department of Earth and Planetary Science, Rutgers University, Piscataway, New Jersey, United States of America; University Copenhagen, Denmark

## Abstract

**Background:**

Despite enormous environmental variability linked to glacial/interglacial climates of the Pleistocene, we have recently shown that marine diatom communities evolved slowly through gradual changes over the past 1.5 million years. Identifying the causes of this ecological stability is key for understanding the mechanisms that control the tempo and mode of community evolution.

**Methodology/Principal Findings:**

If community assembly were controlled by local environmental selection rather than dispersal, environmental perturbations would change community composition, yet, this could revert once environmental conditions returned to previous-like states. We analyzed phytoplankton community composition across >10^4^ km latitudinal transects in the Atlantic Ocean and show that local environmental selection of broadly dispersed species primarily controls community structure. Consistent with these results, three independent fossil records of marine diatoms over the past 250,000 years show cycles of community departure and recovery tightly synchronized with the temporal variations in Earth's climate.

**Conclusions/Significance:**

Changes in habitat conditions dramatically alter community structure, yet, we conclude that the high dispersal of marine planktonic microbes erases the legacy of past environmental conditions, thereby decreasing the tempo of community evolution.

## Introduction

Environmental variability and historical contingencies shape ecosystems by controlling the spatial distribution of species, promoting biological innovation and extinction and, ultimately, driving the evolution of communities [1]–[3]. Ecological theory has yielded two main classes of mechanisms to account for patterns of biodiversity and community assembly: 1) limited dispersal of species combined with unrestricted entry into communities (dispersal-assembly models) [4], and 2) species' dispersal combined with environmental filtering (niche-assembly models) [5], [6]. Dispersal-assembly models predict a progressive decay of community similarity in space and through time, reflecting the effect of dispersal limitation and the stochastic replacement of individuals from the community. By contrast, niche-assembly models are expected to have more predictable community composition among sites and/or time periods characterized by similar environmental conditions [6]. In between these two extreme scenarios, high dispersal rates characteristic of organisms such as marine microbial plankton may potentially overwhelm the effect of spatial constraints and environmental determinants, giving rise to random species' distributions (‘everything is everywhere’) [7]. The ‘everything is everywhere’ hypothesis implies a lack of biogeographic patterns, and, over the last decade, has been a subject of intense debate among aquatic microbial ecologists [7]–[9]. Overall, these ecological theories may help to explain the patterns of biodiversity and community structure observed in the fossil record [2].

The activity of marine phytoplankton, unicellular photoautotrophs that drift with ocean currents, accounts for approximately half of primary production on Earth and sustains marine food webs [10], [11]. Recent work shows that local communities of marine diatoms, a prominent group of phytoplankton in the modern ocean, evolved slowly through gradual changes over the past 1.5 million years (My) of Earth's history [9]. This observation is somewhat surprising because this geological period has witnessed dramatic climate perturbations [12], potentially increasing the rate of community turnover through changes in species' distribution ranges and extinction [13]–[15]. It has been hypothesized that the high dispersal of marine phytoplankton would have ensured species' survival and community recovery [9]; dispersal allows species to track changes in environmental conditions and decreases the probability of extinction [7], [16]. However, our limited understanding of the mechanisms that control the assembly of microbial plankton communities, and the low temporal resolution of the fossil records analyzed to date have precluded testing this hypothesis. Here, we tested this ‘high dispersal-community recovery’ hypothesis by analyzing contemporary phytoplankton communities' structure and fossil records of marine diatoms over the past 250,000 years across large latitudinal gradients in the Atlantic Ocean.

## Methods

### Analysis of modern phytoplankton communities

**Figure 1:**
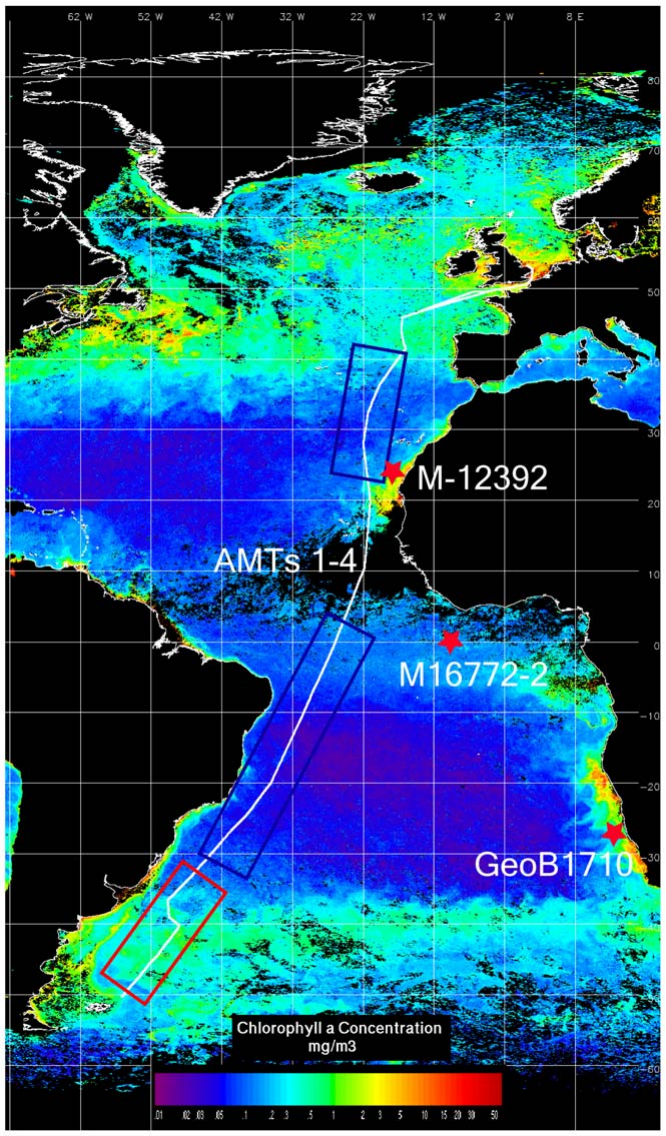
Map showing the sampling sites overlain on a satellite image of ocean color. Atlantic Meridional Transect tracks (white line) separated into subtropical regions (blue boxes) and the sub-Antarctic front (red box), and sediment core sampling sites (red stars).

Data of extant phytoplankton communities were extracted from the Atlantic Meridional Transect (AMT) database (http://web.pml.ac.uk/amt/). The AMT programme offers a unique opportunity to conduct a basin scale study of phytoplankton dynamics across different environmental settings including subtropical gyres, equatorial and coastal upwelling systems, and temperate/subpolar regions. [17]. From September 1995 to May 1997, two meridional transects between Great Britain and the Falkland Islands were carried out each year during the boreal spring and autumn. AMT 1, 2, 3, and 4 were carried out on board RRS James Clark Ross in September–October 1995, April–May 1996, September–October 1996, and April–May 1997, respectively (see Figure 1 for cruise tracks and Table S1 for sampling details). During each cruise, a total of 25 stations were sampled at intervals of approximately 270 nautical miles between 50°N and 50°S. At each station, seawater samples for the determination of chemical and biological variables were collected from 2–5 depths in the upper 200 m of the water column with a set of 12 metal-clean, lever action Teflon Niskin bottles provided with silicone O-rings and seals. Inorganic nutrients were measured colourimetrically in fresh samples using a Technicon AAII Autoanalyser and standard techniques [18]. The detection level was 0.05µM for nitrate and 0.01 µM for phosphate. Duplicate 100-ml seawater samples were preserved, one with 1% buffered formalin (to preserve calcium carbonate structures) and the other with 1% final concentration Lugol's iodine solution. After sedimentation of a subsample for 24 hours (Utermöhl's technique), cells were counted with an inverted microscope and identified to the smallest possible taxonomic level (usually morphospecies level). The volume of water samples used for sedimentation varied between 50 and 256 ml, according to the overall biomass of phytoplankton as shown by fluorometry. The complete database included 360 morphologically defined species belonging to three major taxonomic groups: diatoms, dinoflagellates and coccolithophorids. These phytoplankton groups exhibit striking variations in biomass and species richness along AMT [19], [20]. See Table S2 for a full list of species.

### Fossil records

The data used in this paper were obtained from gravity cores GeoB1710 recovered from the continental slope off Namibia (23.43°S 11.70°E, 2987 m water depth, 1045 cm core length) [21], M16772-2 from the eastern equatorial Atlantic (1°21′S 11°58.4′W, 3913 m water depth) [22], and M12392 from the NW African margin (25°10.3′N 16°50.7′, 2575 m water depth) during R.V. Meteor cruises (Figure 1 and Table S3 for sampling details) [23], [24]. The chronostatigraphy of the cores 16772-2 and M12392 was based on oxygen isotope analyses made on planktonic foraminifera *Globigerinoides sacculifer*. Correlation of the δ^18^O record with the curve of Martinson et al [25] provides chronologic framework prior to 30,000 years ago. For the last 30,000 years, age control was based on Interpolation of U/Th age [26], converted from Accelerator Mass Spectrometry (AMS)-based ^14^C. For core GeoB1710, oxygen isotope analyses of the benthic foraminifera *Cibicidoides wuellerstorfi* provide the basic chronostratigraphic framework. Twenty-six isotopic events were identified between 6000 and 245,000 years ago and correlated with the normalized SPECMAP standard record [27]. The chronostratigraphy of the younger part was based on AMS^14^C dates determined on tests of the planktic foraminifer *Globorotalia inflata* and corrected with −400 years for the apparent age of low-latitude surface ocean water [28].

These sedimentary records were analyzed for diatoms assemblage composition. Microfossil slides were prepared using permanent mounting medium and analyzed using a Nikon microscope with phase-contrast illumination at 1000× magnification [29], [30]. Diatoms were identified at the smallest taxonomic level counting 200–400 specimens (diatom valves) across at least 3 replicate slides of each level. See Table S4 for a full list of species.

The interpretation of diatom assemblages in downcore sediment samples is susceptible to preferential silica preservation/dissolution effects, which could bias the results on community dynamics among the different sampling sites and climatic periods. However, earlier comparisons between living and fossil diatom assemblages along the Portuguese margin and NW African upwelling system have shown that the dominant fossil diatoms in the sediments possess distribution patterns similar to those of their living counterparts in the water column [31]. This indicates that the sedimentary record of diatoms from highly-productive, coastal upwelling regions can be used for paleoecological inferences [31].

Whereas the analysis of extant phytoplankton communities included species of different taxonomic groups (diatoms, dinoflagellates and coccolithophores), our analysis of the fossil record focused exclusively on diatoms. Because of the planktonic life strategy of these organisms in the open ocean, their biogeographic distributions largely are a result of dispersal, and therefore, regardless of the taxonomic groups under consideration, both biogeographic and paleoecological approaches should provide comparable results.

### Species-abundance curves

Preston's representation, displaying the frequency of species across classes of abundance [32], and the rank-abundance species curve, showing the number of individuals versus taxon, with taxa ranked according to their respective abundance, was constructed using the global AMT 1–4 database. Species were sorted into classes of abundance distributed in logarithmic size intervals. Species-abundance curves were constructed for each individual assemblage (sample). Finally, an average species' frequency histogram and rank-abundance species curve were computed.

### Community similarity

The Jaccard index, *J*, a measure of the similarity between communities *j* and *k*, is defined as [33],
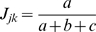
where *a* is the number of species present in both communities *j* and *k*, *b* is the number of species present in community *j*, but absent in *k*, and *c* is the number of species present in community *k*, but absent from *j*. This index requires that species which are jointly absent from *j* and *k* are first removed. *J* ranges from 0 (when no species are shared between any two communities) to 1 (when all species are shared), emphasizes compositional changes, and serves as a metric of β-diversity. Quantitative estimates of community similarity were determined using the Bray-Curtis index (BC), which is defined as,
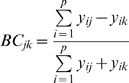
where *y_ij_* and *y_ik_* are the abundances of species (*i = 1, 2, …p*) in samples *j* and *k*. This index emphasizes changes in the most abundant species. Pairwise community similarities were computed using SPSS software.

### Mantel test

To determine the extent to which the spatial distribution of marine phytoplankton assemblages was controlled by local environmental selection or spatial constraints (dispersal limitation), standard and partial Mantel tests were performed using the PASSAGE software for PC [34]. The standard Mantel test is used to compare two independent (dis)similarity matrices describing the same set of entities and to test whether the association is stronger than one would expect from chance [35]. Our null hypothesis predicts that, given the dominance of dispersal, microbial assemblages will be spatially random (“everything is everywhere”). The partial Mantel test is used to determine the relationship between two matrices while holding another one constant which allows separating the effects of spatial constraints from those of environmental controls. Matrices of community similarity were constructed using the Jaccard index between pairs of communities. Environmental matrices were obtained using the coefficient of Euclidean distances for quantitative data (seawater nutrient concentrations, the depth of the nutricline and the nutristad). Other environmental variables such as irradiance and temperature strongly correlate with nutrient availability, and therefore these variables were not included into the analysis. Spatial constraints were calculated as the geographic (Euclidean) distance between each pair of communities.

### Principal component analysis

To further investigate community dynamics through the sedimentary records taking into account species identities, we conducted Principal Component analysis (PCA). This method reduces the data dimensionality by performing a covariance analysis between factors. The procedure transforms a number of possibly correlated variables into a smaller number of uncorrelated factors called principal components. The scores of each sample on the first component (axis) were used to examine changes in community composition through time.

## Results

**Figure 2:**
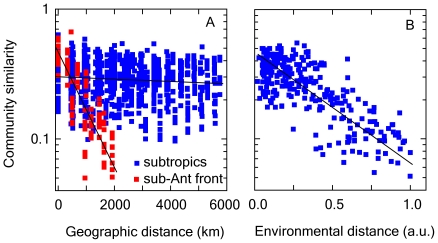
Changes in community similarity across geographic distance and environmental gradients. A. Community similarity vs geographic distance across tropical/subtropical regions and the sub-Antarctic front for Atlantic Meridional Transect (AMT) 3. B. Community similarity vs environmental distance for all data including tropical, subtropical and sub-Antarctic regions of AMT-3. Each point represents a single pair-wise community comparison based on Jaccard similarity index. See Table 1 for statistical results and Figures S1 and S2 for other AMT cruises.

We first compared the taxonomic composition of extant phytoplankton communities including diatoms, dinoflagellates and coccolithophorids using data collected during Atlantic Meridional Transect (AMT) 1–4. The analysis included: i) habitats dominated by oligotrophic conditions extending thousands of kilometers across subtropical and tropical oceans, and ii) habitats characterized by contrasting environmental conditions within nearby oceanic regions across the sub-Antarctic front (Figure 1). Subtropical and tropical ocean systems are characterized by a marked thermal stratification and nutrient depleted surface waters. In contrast, high latitude regions are dominated by strong vertical mixing and high nutrient concentrations throughout the water column. Across the sub-Antarctic front, a sharp increase in chlorophyll *a* concentration highlights a rapid transition from unproductive subtropical waters to highly productive temperate and sub-polar systems (Figure 1). For each individual AMT, community similarity and geographic distance were weakly correlated (data not shown), however, closer inspection of these distance-similarity relationships highlighted two different patterns of community turnover. First, community similarity was not correlated with geographic distance across subtropical/tropical systems (Figure 2A, Table 1 and Figure S1). Second, regardless of the geographic distance, communities assembled under different environmental conditions (e.g. across the sub-Antarctic front) exhibited striking dissimilarities (Figure 2A, Table 1 and Figure S1).

**Table 1:** Statistical parameters for the relationship between community similarity and, geographic distance, and environmental distance.

Region	AMT	slope	intercept	R^2^	*p*
subtropics	1	−5×10^−6^	0.3	0.1	n.s.
(geo distance)	2	−1×10^−5^	0.3	0.04	*
	3	−6×10^−6^	0.3	0.01	*
	4	−5×10^−6^	0.23	0.01	n.s.
sub-ant front	1	−1×10^−4^	0.4	0.77	**
(geo distance)	2	−6×10^−5^	0.32	0.36	**
	3	−2×10^−4^	0.41	0.64	**
	4	−4×10^−4^	0.37	0.81	**
all data	1	−0.31	0.39	0.51	**
(env distance)	2	−0.15	0.3	0.19	**
	3	−0.34	0.39	0.52	**
	4	−0.26	0.35	0.44	**

AMT, Atlantic Meridional Transect. Ordinary least square linear regression model was used to estimate parameters.

** *p*<0.0001, * *p*<0.0005, n.s. no significant.

To quantify the importance of environmental conditions on community structure, we calculated the ‘site-to-site’ environmental distance using five different nutritional variables: nitrate plus nitrite, phosphate and silicate concentration in seawater, the depth of the nutricline and the nutristad (the gradient of nitrate in the nutricline). The two latter parameters can be considered as proxies of nutrient supply to the upper mixed layer of the ocean. Environmental distance explained a significant fraction of community turnover (Figure 2B, Table 1 and Figure S2). The relationship between community similarity and environmental distance is a result of comparing tropical/subtropical waters with highly productive systems characteristic of the sub-Antarctic region. Perhaps surprisingly, Mantel and partial Mantel tests suggest that community similarity was not correlated with environmental variability or geographic distance in the subtropics (Table 2). Across the sub-Antarctic front community similarity was correlated with environmental distance in AMT 3–4, and with geographic distance in AMT 2. Community similarity was correlated with both environmental and geographic distance in AMT 1, but the relationship was not significant when holding matrices constant (Table 2).

**Table 2:** Mantel and partial Mantel test comparisons between community similarity and spatial distribution (i.e., geographic distance between sampling sites), and environmental distance (nutrient availability).

Region	Test	AMT1		AMT2		AMT3		AMT4	
subtropics	E	0.11	n.s.	−0.28	n.s.	0.1	n.s.	−0.14	n.s.
	S	−0.07	n.s.	−0.36	n.s.	−0.07	n.s.	−0.46	**
	E|S	0.11	n.s.	−0.25	n.s.	0.1	n.s.	−0.46	n.s.
	S|E	−0.07	n.s.	−0.3	n.s.	−0.07	n.s.	−0.06	n.s.
sub-ant front	E	−0.64	*	−0.24	n.s.	−0.64	*	−0.68	**
	S	−0.58	*	−0.6	*	−0.4	n.s.	−0.59	**
	E|S	−0.4	n.s.	0.3	n.s.	−0.57	*	−0.4	*
	S|E	−0.2	n.s.	−0.6	*	−0.21	n.s.	0.04	n.s.

AMT, Atlantic Meridional Transect. E, environmental distance; S, spatial distribution; E|S environmental distance holding spatial distribution constant; S|E spatial distribution holding environmental distance constant.

** *p*<0.01, * *p*<0.05, n.s. no significant.

**Figure 3:**
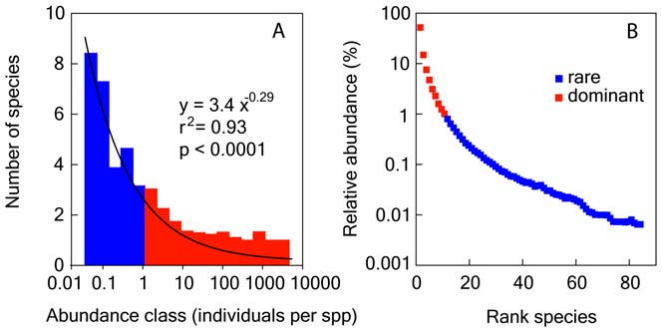
Abundance-dominance curves for marine phytoplankton. A. Preston's representation showing the number of species as a function of population abundance across logarithmic size classes. Black line is the best fit to data. B. Number of individuals versus taxon, with taxa ranked according to their respective abundance. Red and blue squares represent dominant and rare species, respectively. Data from Atlantic Meridional Transect 1–4 were used in these analyses (see Methods for details).

High dispersal implies abundance-dominance relationships characterized by a decreasing power function; i.e., many rare species account for high local diversities and a few dominant taxa form the bulk of community abundance and biomass [36]. On average, the classical Preston's (1960) representation [32], displaying the frequency of species across classes of abundance highlights a high number of species with low population abundances (Figure 3A). Similarly, the number of individuals versus taxon, with taxa ranked according to their respective abundance, further demonstrates the existence of a large pool of rare species with a little contribution to total community abundance (Figure 3B). This ‘seed bank’ (also dubbed ‘rare biosphere’) recruits new species through immigration [36]. But, is there any control on the taxonomic affiliation of immigrants/colonizers?

We assume that, regardless of their taxonomic affiliation, all phytoplankton species reach a given ecosystem periodically (‘everything is everywhere’). However, owing to the low population abundance of rare species, testing this ‘high dispersal’ hypothesis requires analyzing unpractical sample sizes. To circumvent this limitation, we listed species' presence/absence in both subtropical/tropical and sub-Antarctic Polar regions considering the ensemble of samples for each particular region. Assuming that environmental conditions in these ocean regions remained unchanged throughout the period of study, our approach allowed us to increase the area (sample volume) under consideration ∼100-fold (i.e., ∼25 litre). The results of this analysis reveal that these contrasting ocean environments share ∼76% of their total species pool; a significant fraction considering that our method almost certainly still under-samples rare species.

**Figure 4:**
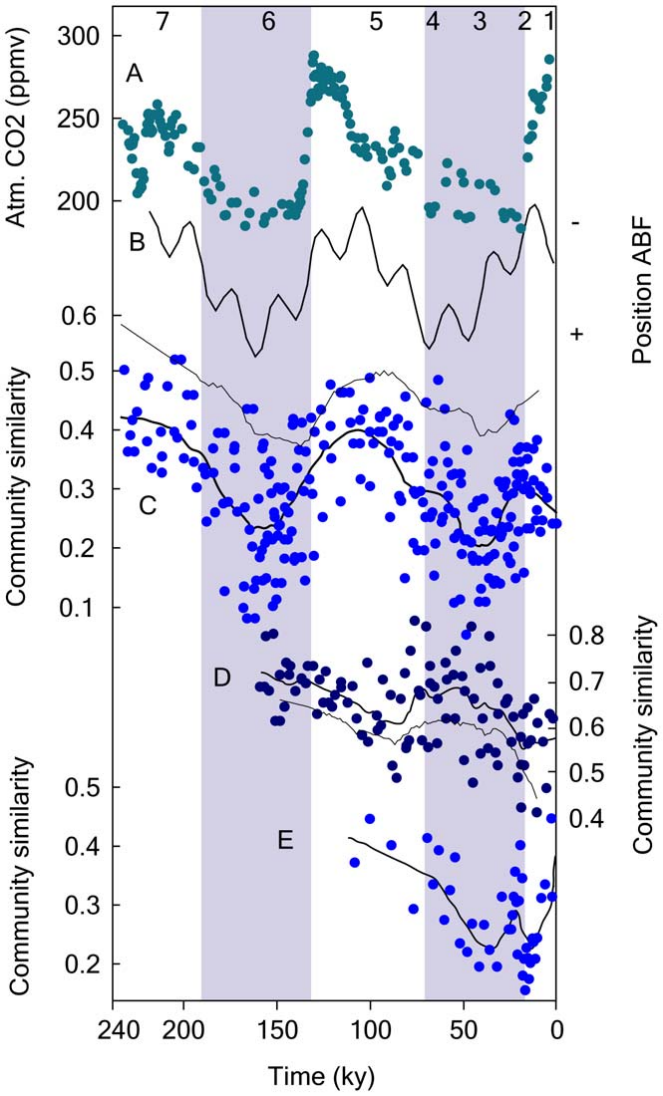
Response of marine diatom communities to past climate change. A. Changes in atmospheric CO_2_ concentration from Vostok ice core [12]. B. Position of the Angola-Benguela front (ABF) indicating variations in the strength of the upwelling [51]. C–E. Jaccard similarity index comparing community composition at each time with the earliest communities of the record in the Walvis basin, SW Africa (GeoB1710), the Eastern Equatorial Atlantic (M16772-2), and the Mauritanian upwelling system (M12392), respectively. Each point is a pair-wise comparison between pair of communities separated in time. Thick and thin lines are the average trend of community similarity calculated using the Jaccard index and the Bray-Curtis quantitative index, respectively. The analysis shows cycles of community departure and recovery. Lower similarity values correspond with comparisons between communities from different climatic periods, and viceversa. Numbers at the top are oxygen isotopic stages. Shaded areas represent glacial periods.

Over the past 250,000 years, Earth's climate has undergone profound and cyclical changes (i.e., glacial/interglacial episodes with 10^4^- to 10^5^-year cyclicity) [12], which offers an excellent framework to study the dynamics of microbial plankton communities through long-term climate perturbations. We analyzed the taxonomic composition of fossil diatom assemblages through time using three independent sedimentary records along the western margin of Africa and the equatorial Atlantic. These regions are strongly influenced by atmospheric forcing, which controls surface ocean circulation, the position and strength of oceanic fronts, and the intrusion of nutrient-rich deep waters into the photic layer (Figure 4A, B). The Jaccard similarity index, comparing community composition at each time with the earliest communities of the record, was plotted against chronological time. Consistent with an equilibrium ecosystem model, our results highlight striking cycles of community departure and recovery tightly coincident with the temporal evolution of Earth's climate, that is atmospheric CO_2_ concentration and atmosphere/ocean physical forcing (Figure 4C–E). This pattern occurred in the three sedimentary records analyzed. To further explore community dynamics taking into account species identities, we conducted a Principal Component Analysis (PCA). The score of each sample on the first axis of the PCA was plotted against time. The analysis shows that community recovery largely was associated with species survival and reassembly (Figure S3). Interestingly, in the case of core GeoB1710, closer inspection of the data revealed higher diversity values during low productive, interglacials (Figure S4).

## Discussion

The evolution of Earth ecosystems and global climate are largely dependent on the origin, maintenance and extinction of biological units that regulate the distribution and cycling of elements, and maintain a self-perpetuating elemental network [37]. The open ocean is recognized as one of the most important and active compartments for biogeochemical cycles, yet, the biogeographic controls and evolutionary mechanisms characterizing the pelagic realm are largely unknown [8], [38], [39]. The geographic distribution of biological species informs on the importance of dispersal, environmental selection and historical contingency in controlling community assembly, and may help to understand the response of marine microbial plankton communities to climate change.

The biogeographic patterns delineated here are associated with sharp environmental gradients such as those located across the sub-Antarctic front. Other oceanographic structures such as the equatorial divergence or the north temperate-subtropical front can potentially influence phytoplankton community structure across the AMT. However, we focused on the sub-Antarctic front as previous work has revealed marked changes in phytoplankton biomass and primary productivity across this oceanic region [19]. The biogeographic distributions reported here could be associated with the existence of physical barriers such as water mass fronts, limiting habitat connectivity and species' dispersal ranges. Indeed, the geographic isolation of biological units mediated by thermal and salinity gradients has been proposed as a mechanism promoting allopatric speciation in the ocean [40]. If these physical barriers effectively limited microbial plankton dispersal, we would expect to find biogeographic differences between tropical/subtropical regions to the north and south of the equatorial divergence. However, communities inhabiting these oceanic environments exhibited striking similarities (Figure 2A and Table 2).

Much evidence indicates that marine microbial morphospecies consist of a number of cryptic species with subtle morphological differences. Several studies have shown that these cryptic species are adapted to particular ecological niches [38]. This could constitute an evolutionary strategy to increase the geographic distribution ranges and buffer species against extinction. Including the diversity of cryptic species in our analysis, changes in community similarity across environmental gradients would have been even larger than those reported here. The extent to which the spatial distribution of these cryptic species could be controlled by spatial constraints is not straightforward, however, previous work has shown that, although disjunct, cryptic forms of the diatom *Skeletonema* possess world-wide distribution [41].

Biotic interactions between microbial plankton species emerge and disappear in the order of days to weeks in response to environmental variability [42]. Often the whole community changes due to environmental reset. Their high growth/loss rates (∼four orders of magnitude higher than those of forest trees) and planktonic nature (i.e., phytoplankton drift with ocean currents), are likely to be responsible for the ephemeral dominance and rapid succession of microbial plankton species in the ocean [43], [44]. Arguably, these biological communities lack of an evolutionarily acquired network of biotic interactions. But is their low resistance to environmental change synonymous of community fragility?

In a previous report [9], we have shown that local communities of marine diatoms evolved slowly through gradual changes over the past 1.5 million years. However, the low temporal resolution of the fossil records analyzed (tens to hundreds of thousands of years) precluded obtaining a more detailed picture of the effect of climate change on community dynamics. Here, using fossil records with a higher temporal resolution, we show that diatom biodiversity and community structure largely recovered from dramatic climate perturbations in the past. The strong correlation between atmospheric CO_2_ levels (from Antarctic ice cores) and community dynamics is somewhat surprising taking into account that, in addition to changes in global climate, the fossil diatom assemblages analyzed here were influenced by local determinants such as regional current systems, terrestrial nutrient inputs, atmospheric deposition, physical mixing, etc [31], [45]. Furthermore, it is well known that there are substantial differences between subsequent glacial and interglacial stages and previous evidence indicates that non-analog climates produce non-analog community assemblages.

Similar results have been reported for other microbial plankton groups during different periods of Earth's climatic history. Recent evidence shows that the vast majority of calcareous nanoplankton, including coccolithophores and foraminifera, survived across the Paleocene/Eocene thermal maximum event (∼55 million years ago) [46], an episode characterized by rapidly rising atmospheric CO_2_, global warming and ocean acidification [47]. Furthermore, the fossil record has repeatedly shown that microbial plankton species track changes in environmental conditions [14], which, in conjunction with their broad dispersal ranges, allows habitat re-colonization and community recovery.

The broad dispersal of marine planktonic microbes contrasts with the limited dispersal ranges of marine and terrestrial plants and animals. Dispersal limitation increases species' vulnerability to climate change and habitat fragmentation, which, however, exert a minor impact on the distribution of species with global dispersal ranges. These fundamental differences between marine planktonic microbes and macroorganisms suggest different patterns of community evolution.

Our results provide an explanation for the slow and gradual evolution of marine diatom communities across the Pleistocene [9]. Environmental changes rapidly alter community structure, yet, the great potential for dispersal of microbial plankton species confer planktonic ecosystems the ability to hold in check every taxonomic unit required to ensure community recovery. We conclude that marine phytoplankton communities are generally robust with respect to species composition over geological time scales on order of 1 million years. However, this assertion raises important questions concerning the mechanisms that control speciation, extinction and community turnover in marine microbial plankton [48]–[50]. What causes the extinction of species with global dispersal ranges? What controls the tempo and mode of community evolution? Our analysis provides some clues such that glacial/interglacial climatic cycles might play a minor role on long-term community evolution. Currently, the extent to which extrinsic factors such as climate change or biotic pressures such as resource competition dominate the evolution of marine microbial plankton remains uncertain. Understanding these fundamental questions must be key to aquatic microbial ecologists and demands further integration of the fields of molecular biology, evolutionary ecology and micropaleontology.

## Supporting Information

Figure S1 Relationship between community similarity and geographic distance across subtropical regions and the sub-Antarctic front for Atlantic Meridional Transect 1–4. See Table 1 for statistical parameters.
(0.87 MB TIF)

Figure S2 Relationship between community similarity and environmental distance for Atlantic Meridional Transect 1–4. Only samples collected at surface were used in these analyses. See Table 1 for statistical parameters.
(0.76 MB TIF)

Figure S3 Jaccard similarity index and the score of the sample on the first component of a Principal Component Analysis (PCA) against chronological time. Blue line is the average trend of community similarity calculated using the Jaccard index. Dots are the score of each sample on the first axis of the PCA.
(0.76 MB TIF)

Figure S4 Changes in diatom species richness along sedimentary records.
(0.85 MB TIF)

Table S1 Sampling details for Atlantic Meridional Transects 1–4.
(0.20 MB DOC)

Table S2 List of phytoplankton species identified during Atlantic Meridional Transects 1–4.
(0.15 MB DOC)

Table S3 Geographic coordinates, sample depth and age for diatom fossil records.
(0.11 MB DOC)

Table S4 Diatom species list in sedimentary records.
(0.06 MB DOC)
