# Supplementary figures and images for: Phytoplankton Biogeography and Community Stability in the Ocean
Source: PLoS One. 2010 Apr 2;5(4):e10037. doi: 10.1371/journal.pone.0010037 (PMC2848864; doi:10.1371/journal.pone.0010037)

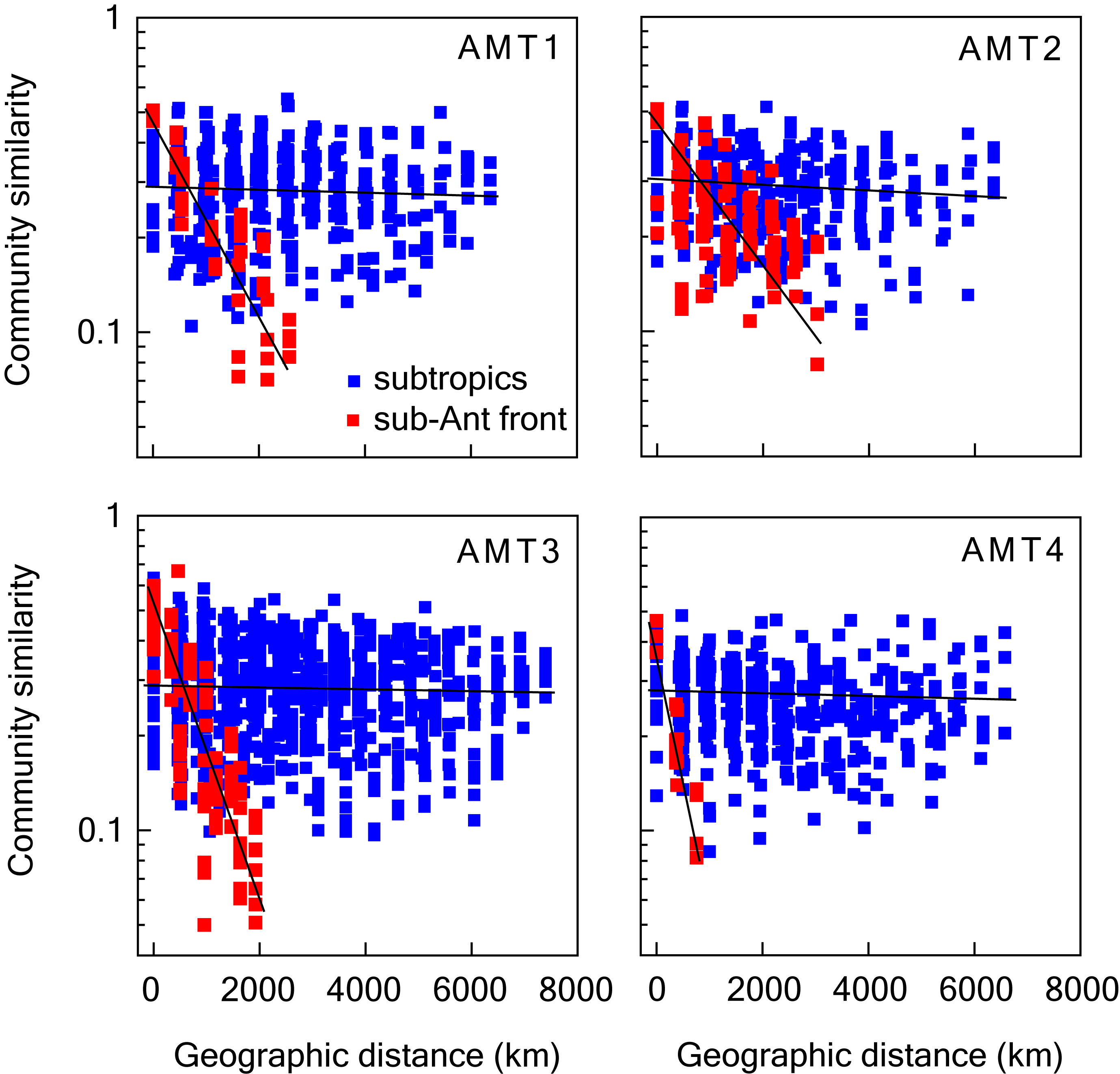

Supplement: Figure S1 — Relationship between community similarity and geographic distance across subtropical regions and the sub-Antarctic front for Atlantic Meridional Transect 1–4. See Table 1 for statistical parameters. (0.87 MB TIF) [file pone.0010037.s001.tif]

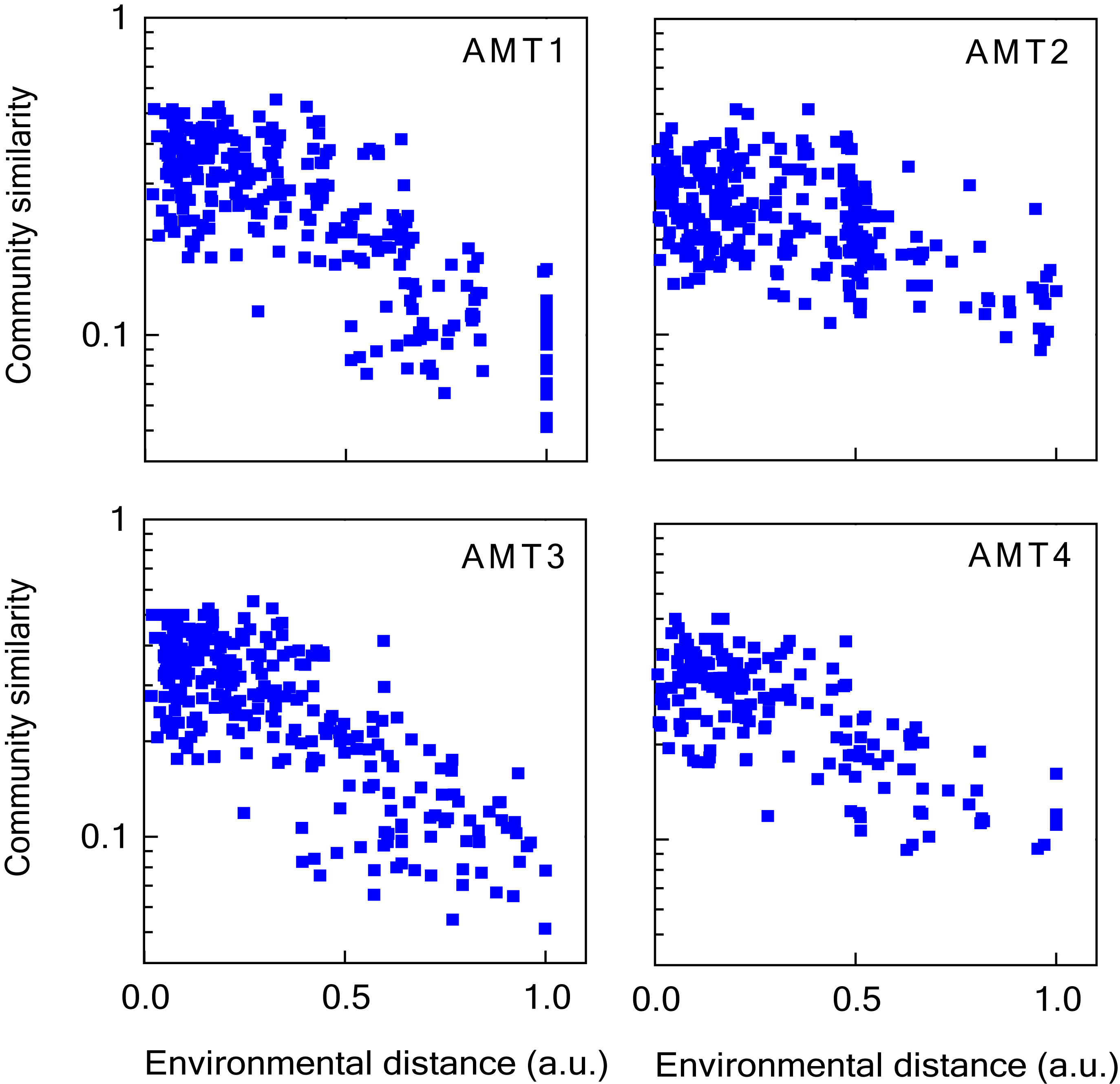

Supplement: Figure S2 — Relationship between community similarity and environmental distance for Atlantic Meridional Transect 1–4. Only samples collected at surface were used in these analyses. See Table 1 for statistical parameters. (0.76 MB TIF) [file pone.0010037.s002.tif]

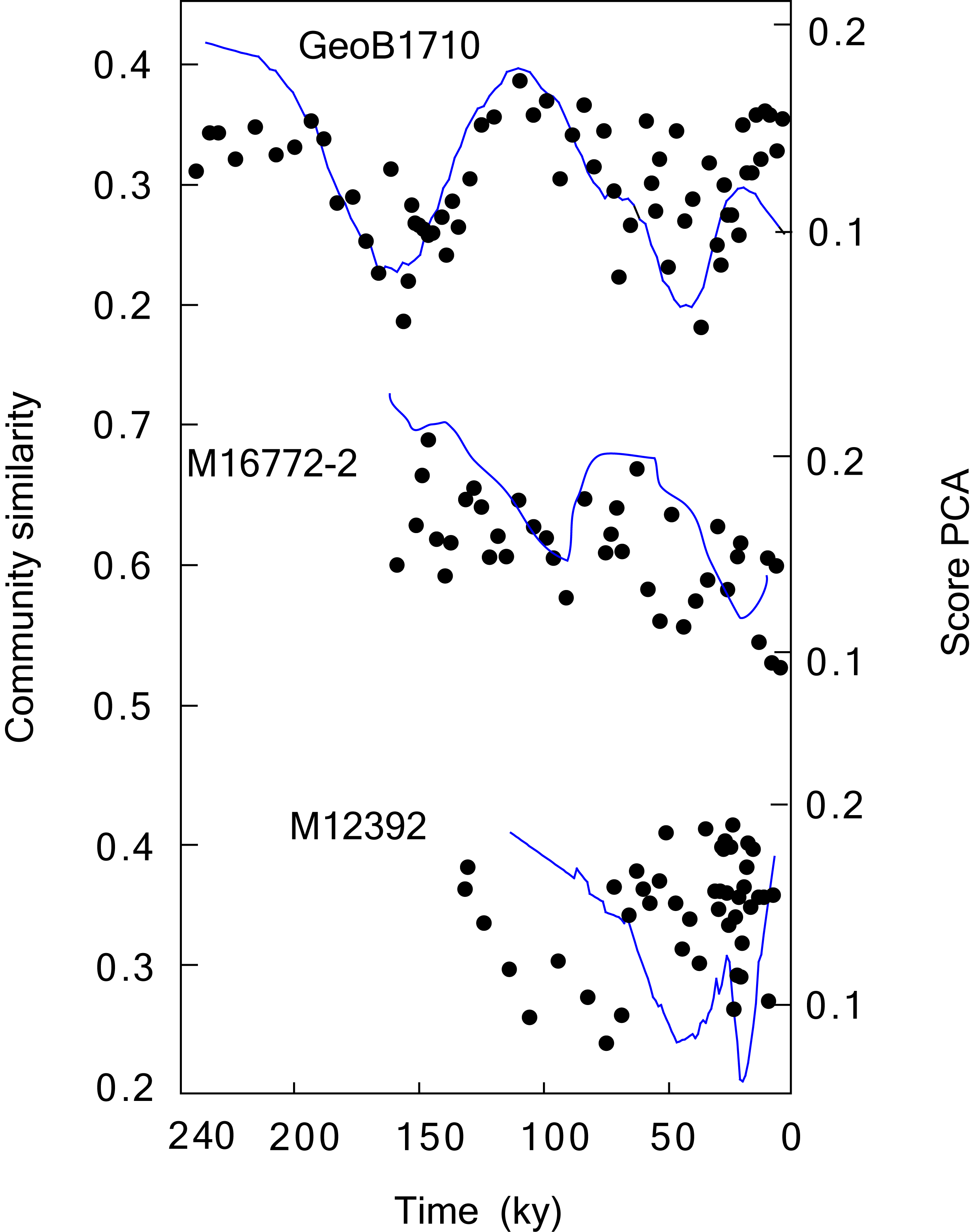

Supplement: Figure S3 — Jaccard similarity index and the score of the sample on the first component of a Principal Component Analysis (PCA) against chronological time. Blue line is the average trend of community similarity calculated using the Jaccard index. Dots are the score of each sample on the first axis of the PCA. (0.76 MB TIF) [file pone.0010037.s003.tif]

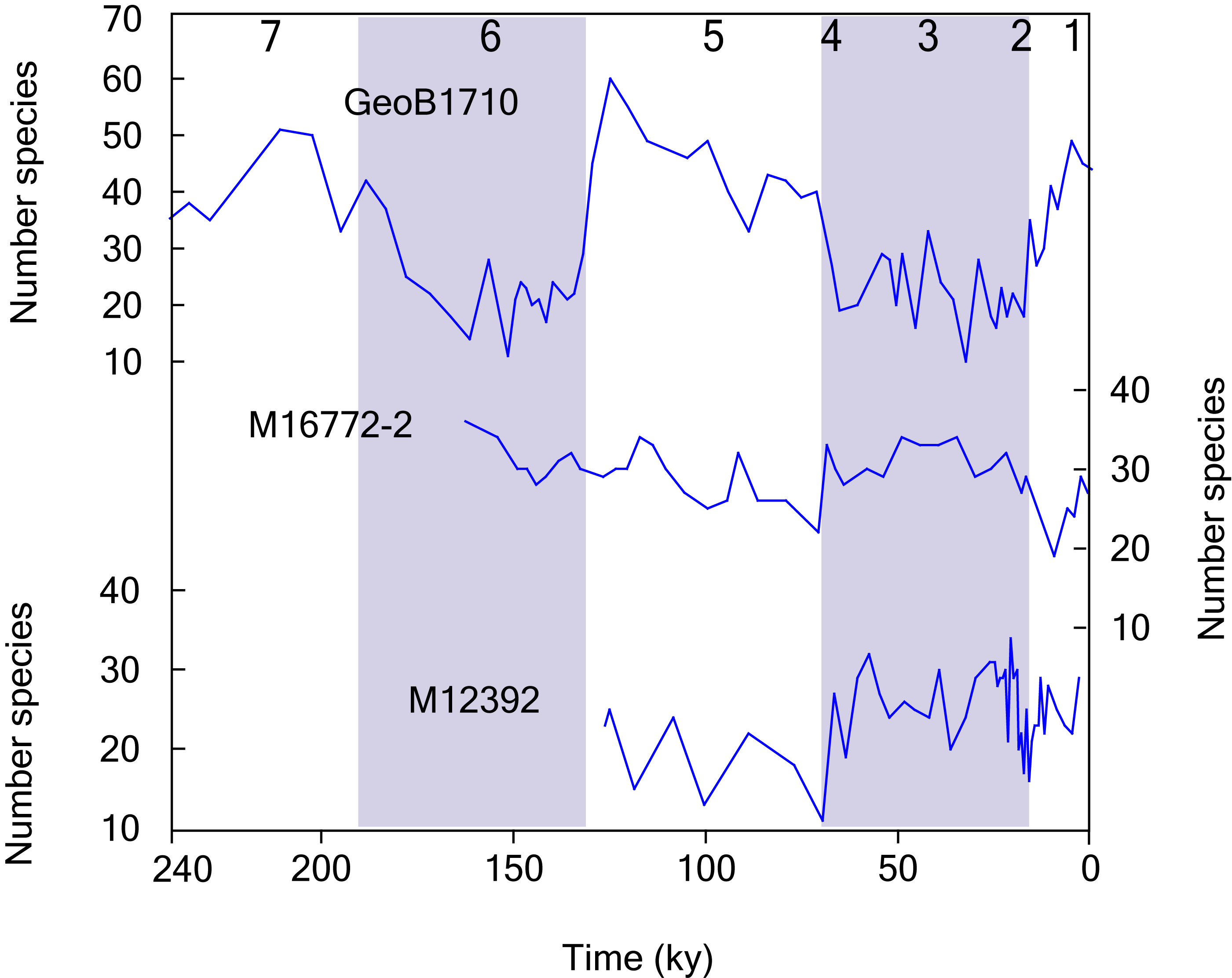

Supplement: Figure S4 — Changes in diatom species richness along sedimentary records. (0.85 MB TIF) [file pone.0010037.s004.tif]
