# Supplementary material for: Phytoplankton Biogeography and Community Stability in the Ocean
Source: PLoS One. 2010 Apr 2;5(4):e10037. doi: 10.1371/journal.pone.0010037 (PMC2848864; doi:10.1371/journal.pone.0010037)
Supplement: Table S1 — Sampling details for Atlantic Meridional Transects 1–4. (0.20 MB DOC) [file pone.0010037.s005.doc]

| Date | Time (GMT) | Latitude  (+ve deg. N) | Longitude (+ve deg. E) | Sampling depth (m) |
| --- | --- | --- | --- | --- |
|  |  |  |  |  |
| 25/09/1995 | 12:46 | 48.9 | -9.1 | 7 |
|  |  |  |  | 50 |
| 26/09/1995 | 12:38 | 47.9 | -14.9 | 7 |
|  |  |  |  | 40 |
| 27/09/1995 | 11:00 | 47.0 | -20.0 | 7 |
|  |  |  |  | 50 |
| 28/09/1995 | 13:39 | 42.3 | -20.0 | 7 |
|  |  |  |  | 60 |
| 29/09/1995 | 13:24 | 37.9 | -20.0 | 7 |
|  |  |  |  | 90 |
| 30/09/1995 | 13:25 | 33.6 | -20.9 | 7 |
|  |  |  |  | 110 |
| 2/10/1995 | 14:34 | 27.4 | -21.8 | 7 |
|  |  |  |  | 80 |
| 3/10/1995 | 13:16 | 23.3 | -21.2 | 7 |
|  |  |  |  | 80 |
| 4/10/1995 | 11:23 | 19.5 | -20.4 | 7 |
|  |  |  |  | 30 |
| 5/10/1995 | 16:24 | 13.0 | -20.4 | 7 |
|  |  |  |  | 70 |
| 6/10/1995 | 14:21 | 9.2 | -22.2 | 7 |
|  |  |  |  | 60 |
| 7/10/1995 | 14:17 | 5.4 | -24.0 | 7 |
|  |  |  |  | 80 |
| 8/10/1995 | 14:16 | 1.4 | -25.7 | 7 |
|  |  |  |  | 80 |
| 9/10/1995 | 14:17 | -2.8 | -27.5 | 7 |
|  |  |  |  | 90 |
| 10/10/1995 |  |  |  | 7 |
|  |  |  |  | 100 |
| 11/10/1995 | 15:35 | -11.5 | -31.6 | 7 |
|  |  |  |  | 140 |
| 12/10/1995 | 15:24 | -15.6 | -33.4 | 7 |
|  |  |  |  | 150 |
| 13/10/1995 | 15:25 | -19.6 | -35.4 | 7 |
|  |  |  |  | 160 |
| 14/10/1995 | 15:24 | -23.8 | -37.4 | 7 |
|  |  |  |  | 120 |
| 15/10/1995 | 15:19 | -27.1 | -40.1 | 7 |
|  |  |  |  | 80 |
| 16/10/1995 | 15:19 | -30.3 | -43.4 | 7 |
|  |  |  |  | 100 |
| 17/10/1995 | 15:23 | -33.2 | -46.4 | 7 |
|  |  |  |  | 40 |
| 22/10/1995 | 17:38 | -41.0 | -55.3 | 7 |
|  |  |  |  | 40 |
| 23/10/1995 | 16:22 | -46.0 | -56.3 | 7 |
|  |  |  |  | 40 |
| 24/10/1995 | 14:18 | -50.8 | -57.4 | 7 |
|  |  |  |  | 30 |
| 23/04/1996 | 14:20 | -47.6 | -55.6 | 7 |
|  |  |  |  | 60 |
| 24/04/1996 | 13:56 | -43.4 | -54.7 | 7 |
|  |  |  |  | 30 |
| 25/04/1996 | 14:08 | -39.4 | -53.3 | 7 |
|  |  |  |  | 50 |
| 29/04/1996 | 17:13 | -36.1 | -49.8 | 7 |
|  |  |  |  | 80 |
| 30/04/1996 | 13:17 | -33.4 | -46.6 | 7 |
|  |  |  |  | 70 |
| 1/5/1996 | 13:11 | -30.3 | -43.4 | 7 |
|  |  |  |  | 110 |
| 2/5/1996 | 13:06 | -27.6 | -40.7 | 7 |
|  |  |  |  | 80 |
| 3/5/1996 | 12:23 | -24.4 | -37.9 | 7 |
|  |  |  |  | 100 |
| 4/5/1996 | 12:02 | -19.9 | -35.5 | 7 |
|  |  |  |  | 7 |
| 5/5/1996 | 12:11 | -15.2 | -33.3 | 150 |
|  |  |  |  | 7 |
| 6/5/1996 | 12:09 | -11.2 | -31.5 | 70 |
|  |  |  |  | 130 |
| 7/5/1996 | 12:03 | -7.5 | -29.8 | 7 |
|  |  |  |  | 30 |
|  |  |  |  | 100 |
| 8/5/1996 | 11:02 | -3.9 | -28.2 | 7 |
|  |  |  |  | 60 |
| 9/5/1996 | 11:05 | -0.2 | -26.4 | 7 |
|  |  |  |  | 40 |
|  |  |  |  | 70 |
| 10/5/1996 | 11:08 | 3.4 | -24.8 | 7 |
|  |  |  |  | 60 |
| 11/5/1996 | 11:03 | 7.6 | -22.9 | 7 |
|  |  |  |  | 50 |
| 12/5/1996 | 11:00 | 11.7 | -21.1 | 7 |
|  |  |  |  | 40 |
| 14/05/1996 | 11:03 | 21.8 | -21.5 | 7 |
|  |  |  |  | 70 |
| 15/05/1996 | 9:58 | 26.5 | -21.8 | 7 |
|  |  |  |  | 90 |
| 16/05/1996 | 10:02 | 30.9 | -21.3 | 7 |
|  |  |  |  | 80 |
| 17/05/1996 | 10:00 | 35.7 | -20.4 | 7 |
|  |  |  |  | 60 |
| 18/05/1996 | 10:00 | 39.9 | -20.0 | 7 |
|  |  |  |  | 40 |
| 19/05/1996 | 10:00 | 44.1 | -20.0 | 7 |
|  |  |  |  | 50 |
| 20/05/1996 | 9:04 | 46.5 | -14.1 | 7 |
|  |  |  |  | 20 |
| 21/05/1996 | 9:03 | 48.8 | -8.1 | 7 |
|  |  |  |  | 30 |
| 24/09/1996 | 10:38 | 47.4 | -18.2 | 7 |
|  |  |  |  | 40 |
| 25/09/1996 | 10:38 | 42.9 | -20.0 | 7 |
|  |  |  |  | 40 |
| 26/09/1996 | 11:38 | 38.2 | -20.0 | 7 |
|  |  |  |  | 40 |
|  |  |  |  | 80 |
| 27/09/1996 | 11:29 | 34.0 | -21.3 | 7 |
|  |  |  |  | 80 |
| 28/09/1996 | 11:39 | 29.5 | -21.8 | 7 |
|  |  |  |  | 110 |
| 29/09/1996 | 11:31 | 24.7 | -21.4 | 7 |
|  |  |  |  | 40 |
|  |  |  |  | 90 |
| 30/09/1996 | 14:52 | 20.1 | -20.6 | 7 |
|  |  |  |  | 20 |
|  |  |  |  | 40 |
| 2/10/1996 | 11:05 | 12.8 | -20.5 | 7 |
|  |  |  |  | 20 |
|  |  |  |  | 20 |
| 3/10/1996 | 11:02 | 9.1 | -22.3 | 7 |
|  |  |  |  | 30 |
|  |  |  |  | 50 |
| 4/10/1996 | 11:05 | 5.2 | -24.0 | 7 |
|  |  |  |  | 40 |
|  |  |  |  | 70 |
| 5/10/1996 | 11:02 | 1.3 | -25.8 | 7 |
|  |  |  |  | 40 |
|  |  |  |  | 80 |
| 6/10/1996 | 11:02 | -2.4 | -27.5 | 7 |
|  |  |  |  | 40 |
|  |  |  |  | 85 |
| 7/10/1996 | 11:03 | -6.5 | -29.3 | 7 |
|  |  |  |  | 50 |
|  |  |  |  | 110 |
| 8/10/1996 | 12:00 | -10.8 | -31.2 | 7 |
|  |  |  |  | 75 |
|  |  |  |  | 130 |
| 9/10/1996 | 12:21 | -14.9 | -33.1 | 7 |
|  |  |  |  | 60 |
|  |  |  |  | 140 |
| 10/10/1996 | 12:04 | -18.9 | -35.0 | 7 |
|  |  |  |  | 75 |
|  |  |  |  | 125 |
| 11/10/1996 | 12:21 | -22.9 | -37.0 | 7 |
|  |  |  |  | 60 |
|  |  |  |  | 100 |
| 12/10/1996 | 12:01 | -26.6 | -39.6 | 7 |
|  |  |  |  | 95 |
| 13/10/1996 | 12:01 | -29.9 | -42.9 | 7 |
|  |  |  |  | 50 |
|  |  |  |  | 90 |
| 14/10/1996 | 12:02 | -32.8 | -46.1 | 7 |
|  |  |  |  | 40 |
|  |  |  |  | 60 |
| 15/10/1996 | 12:44 | -35.7 | -49.6 | 7 |
|  |  |  |  | 30 |
|  |  |  |  | 50 |
| 16/10/1996 | 11:56 | -37.8 | -52.2 | 7 |
|  |  |  |  | 40 |
|  |  |  |  | 60 |
| 23/10/1996 | 13:11 | -43.6 | -55.0 | 7 |
|  |  |  |  | 30 |
|  |  |  |  | 50 |
| 24/10/1996 | 13:05 | -48.0 | -55.9 | 7 |
|  |  |  |  | 40 |
|  |  |  |  | 80 |
| 25/10/1996 | 11:07 | -51.9 | -57.9 | 7 |
|  |  |  |  | 20 |
|  |  |  |  | 40 |
| 21/04/1997 | 17:23 | -51.0 | -57.3 | 7 |
|  |  |  |  | 80 |
| 22/04/1997 | 14:08 | -47.6 | -55.9 | 7 |
|  |  |  |  | 50 |
| 23/04/1997 | 14:02 | -43.5 | -54.4 | 7 |
|  |  |  |  | 50 |
| 30/04/1997 | 13:40 | -35.7 | -49.8 | 7 |
|  |  |  |  | 20 |
| 1/5/1997 | 13:14 | -32.6 | -46.2 | 7 |
|  |  |  |  | 50 |
| 2/5/1997 | 13:09 | -29.3 | -42.7 | 7 |
|  |  |  |  | 80 |
| 3/5/1997 | 12:58 | -26.1 | -39.2 | 7 |
|  |  |  |  | 80 |
| 4/5/1997 | 12:56 | -22.2 | -36.6 | 7 |
|  |  |  |  | 110 |
| 5/5/1997 | 11:56 | -18.3 | -34.8 | 7 |
|  |  |  |  | 40 |
| 6/5/1997 | 11:57 | -14.1 | -32.8 | 7 |
|  |  |  |  | 130 |
| 7/5/1997 | 11:58 | -10.0 | -30.9 | 7 |
|  |  |  |  | 120 |
| 8/5/1997 | 11:57 | -5.9 | -29.1 | 7 |
|  |  |  |  | 110 |
| 9/5/1997 | 11:56 | -2.0 | -27.3 | 7 |
|  |  |  |  | 70 |
| 10/5/1997 | 11:57 | 1.9 | -25.6 | 7 |
|  |  |  |  | 72 |
| 11/5/1997 | 11:58 | 6.1 | -23.6 | 7 |
|  |  |  |  | 40 |
| 12/5/1997 | 12:05 | 10.1 | -21.9 | 7 |
|  |  |  |  | 60 |
| 13/05/1997 | 11:55 | 13.8 | -21.0 | 7 |
|  |  |  |  | 30 |
| 14/05/1997 | 10:56 | 17.8 | -21.2 | 7 |
|  |  |  |  | 60 |
| 15/05/1997 | 10:55 | 22.2 | -21.6 | 7 |
|  |  |  |  | 40 |
| 16/05/1997 | 10:59 | 26.3 | -21.9 | 7 |
|  |  |  |  | 80 |
| 17/05/1997 | 10:54 | 30.0 | -21.8 | 7 |
| 18/05/1997 | 10:55 | 33.7 | -21.3 | 7 |
|  |  |  |  | 90 |
| 19/05/1997 | 10:54 | 36.2 | -20.8 | 7 |
|  |  |  |  | 80 |
| 20/05/1997 | 10:57 | 39.9 | -20.0 | 7 |
| 21/05/1997 | 11:04 | 44.0 | -20.0 | 7 |
|  |  |  |  | 25 |
| 22/05/1997 | 10:04 | 47.0 | -20.0 | 15 |
| 24/05/1997 | 10:01 | 49.0 | -8.8 | 7 |
|  |  |  |  | 20 |

Table S1. Sampling details for Atlantic Meridional Transects 1-4
