# Supplementary material for: Phytoplankton Biogeography and Community Stability in the Ocean
Source: PLoS One. 2010 Apr 2;5(4):e10037. doi: 10.1371/journal.pone.0010037 (PMC2848864; doi:10.1371/journal.pone.0010037)
Supplement: Table S2 — List of phytoplankton species identified during Atlantic Meridional Transects 1–4. (0.15 MB DOC) [file pone.0010037.s006.doc]

| Table S2. List of phytoplankton species identified during Atlantic Meridional Transects 1-4. | | |
| --- | --- | --- |
|  |  |  |
| DIATOMS |  |  |
| *Actinocyclus sp* | *Chaetoceros saltans* | *Navicula cf planamembranacea* |
| *Amphora sp.* | *Chaetoceros similis* | *Navicula septentrionalis* |
| *Asterolampra sp.* | *Chaetoceros simplex* | *Nitzschia sp. A cf F. oceanica* |
| *Asteromphalus cf. heptactis* | *Chaetoceros sociale* | *Nitzschia sp.B cf. F kergulensis* |
| *Asteromphalus* 60µm | *Chaetoceros teres* | *Colonial Nitzschia sp.C* |
| *Asteromphalus sarcophagus* | *Chaetoceros tetrastichon* | *Colonial Nitzschia sp D cf. F cylindrus* |
| *Asteromphalus cf. wyvilleae* | *Chaetoceros tortissimus* | *Nitzschia bicapitata* |
| *Asterionellopsis bleakeleyi* | *Chaetoceros vixvisiblis* | *Nitzschia 'closterium'* |
| *Bacteriastrum biconicum* | *Climacodium frauenfeldii* | *Nitzschia 'delicatissima'* |
| *Bacteriastrum comosum* | *Climacosphenia sp.* | *Nitzschia 'seriata'* |
| *Bacteriastrum elongatum* | *Corethron criophillum* 5µm | *Nitzschia panduriformis* |
| *Bacteriastrum furcatum* | *Corethron criophillum* 15µm | *Nitzschia* 250µm |
| *Bacteriastrum hyalinum* | *Corethron criophillum* 30µm | *Nitzschia* 400µm |
| *Bacteriastrum 'solitarium'* | *Corethron inermis* | *Odontella weissflogii* |
| *Bacteriosira fragilis* | *Coscinodiscus africanus* | *Plagiotropis sp.* |
| *Bellarochia malleus* | *Coscinodiscus asteromphalus* | *Paralia sulcata* |
| *Cerataulina pelagica* | *Coscinodiscus radiatus* | *Planktoniella sol* |
| *Cerataulina sp.* | *Coscinodiscus cf marginatus* | *Pleurosigma sp.* |
| *Chaetoceros sp. a* | *Coscinodiscus cf. argus* | *Pleurosigma planctonicum* |
| *Chaetoceros sp. b* | *Dactyliosolen sp.* | *Podosira stelliger* |
| *Chaetoceros affinis* | *Dactyliosolen antarctica* | *Porosira cf denticulata* |
| *Chaetoceros anastomosans* | *Dactyliosolen phuketensis* | *Phaeodactylum sp* |
| *Chaetoceros atlanticum var.* | *Detonula pumila* | *Pseudotriceratium punctatum* |
| *Chaetoceros atlant. v. neapol.* | *Diatoma elongatus f. tenuis* | *Rhizosolenia alata* 2µm |
| *Chaetoceros atlant. v. skeleton* | *Ditylum brightwellii* | *Rhizosolenia alata* 5µm |
| *Chaetoceros borealis* | *Eucampia antarctica* | *Rhizosolenia alata* 10µm |
| *Chaetoceros brevis* | *Eucampia cornuta* | *Rhizosolenia alata* 15µm |
| *Chaetoceros compressum* | *Eucampia groenlandica* | *Rhizosolenia alata* 25µm |
| *Chaetoceros compressum pa* | *Fragilariopsis sp* | *Rhizosolenia bergonii* |
| *Chaetoceros concavicorne* | *Fragilariopsis doliolus* | *Rhizosolenia calcar-avis* |
| *Chaetoceros convolutum* | *Guinardia flaccida* | *Rhizosolenia chunii* |
| *Chaetoceros curvisetus* | *Guinardia blavyana* | *Rhizosolenia cylindrus* |
| *Chaetoceros dadayi* | *"Guinardia striata"* | *Rhizosolenia delicatula* |
| *Chaetoceros danicus* | *Haslea warwickii* | *Rhizosolenia fragilissima* |
| *Chaetoceros debilis* | *Hemiaulus hauckii* | *Rhizosolenia heb. f. ssp.* 10µm |
| *Chaetoceros decipiens* | *Hemiaulus sinensis* | *Rhizosolenia heb. f. ssp.* 30µm |
| *Chaetoceros densus* | *Hemiaulus membranaceus* | *Rhizosolenia hyalina* |
| *Chaetoceros didymus* | *Lauderia annulata* | *Rhizosolenia inermis* |
| *Chaetoceros diversus* | *Leptocylindrus danicus* | *Rhizosolenia cf pungens* |
| *Chaetoceros laciniosus* | *Leptocylindrus minimus* | *Rhizosolenia robusta* |
| *Chaetoceros messanensis* | *Leptocylindrus mediterranea* | *Rhizosolenia simplex* |
| *Chaetoceros neglectum* | *Lioloma delicatulum* | *Rhizosolenia shrubsolei* 2µm |
| *Chaetoceros neogracilis* | *Lioloma elongatum* | *Rhizosolenia shrubsolei* 5µm |
| *Chaetoceros cf pelagicus* | *Lioloma pacificum* | *Rhizosolenia stolterfothii s* |
| *Chaetoceros pendulus* | *Mastogloia rostrata* | *Rhizosolenia stolterfothii L* |
| *Chaetoceros perpusilis* | *Membraneis sp.* | *Rhizosolenia stolterfothii* 120µm |
| *Chaetoceros peruvianus* | *Pennate* | *Rhizosolenia stolterfothii* 60µm |
| *Chaetoceros protuberans* | *Pennate* Small | *Rhizosolenia styliformis* |
| *Chaetoceros radians* | *Pennate* 50µm | *Roperia tessellata* |
| *Chaetoceros radicans* | *Nanoneis haslea* | *Skeletonema costatum* |
| *Chaetoceros rostratum* | *Navicula directa* | *Stephanopyxix turris* |
| *Stauroneis membranacea* | *Ceratium pulchellum* | *Ceratocorys gorrettii* |
| *Streptotheca sp.* | *Ceratium ranipes* | *Citharistes apsteinii* |
| *Synedra sp.* | *Ceratium teres* | *Colchlodinium sp.* |
| *Synedra hantzschiana* | *Ceratium trichoceros* | *Diplopsalopsis sp* |
| *Thalassionema nitzschiodes* | *Ceratium tripos* | *Dinophysis cf brevisulcus* |
| *Thalassionema bacillaris* | *Ceratium vultur* | *Dinophysis cf exigua* |
| *Thalassionema frauefeldii* | *Dinophysis acuminata* | *Dinophysis cf favus* |
| *Thalassiosira eccentrica* | *Dinophysis caudata* | *Dinophysis lens* |
| *Thalassiosira gravida* | *Dinophysis lens* | *Dinophysis ovum* |
| *Thalassiosira cf. gracilis* | *Dinophysis schuetii* | *Dinophysis cf parvulum* |
| *Thalassiosira cf. 'subtilis'* | *Gonyaulax sp* | *Dinophysis rotundatum* |
| *Centric sp* 4µm | *Gonyaulax digitalis* | *Diplopsalopsis sp* |
| *Centric sp.* 10µm | *Gonyaulax grindleyi* | *Erythropsis sp.* |
| *Centric sp.* 15µm | *Gonyaulax milneri* | *Goniodoma sp.* |
| *Centric sp.* 20µm | *Gonyaulax polygramma* | *Gymnodinium sp.* |
| *Centric sp.* 30µm | *Gonyaulax spinifera* | *Gymnodimium sp.*20µm |
| *Centric sp,* 50µm | *Gymnodinium sp. A* | *Gymnodinioid* |
| *Centric sp,* 60µm | *Gymnodinium cf conicum* | *Hexasterias* |
| *Centric sp,* 70µm | *Gymnodinium cf galeaeforme* | *Rhizomonas. setigera -* Symbiont |
| *Thalassiothrix antarctica* | *Gymnodinium sp* 10µm *B* | *Gyrodinium cf. fusiformis* |
| *Thalassiothrix longissima* | *Gyrodinium* 25µm *cf G. aureolum* | *Gyrodinium cf. brittannicum* |
| *Pseudotriceratium sp.* | *Gyrodinium falcatum* | *Histoneis bipolporoides* |
| *Tropidoneis sp.* | *Goniodoma polyedrica* | *Histoneis hyalina* |
| *Valdiviella formosa* | *Lissodinium sp.* | *Katodinium sp.* |
|  | *Mesoporos perforatus* | *Katodinium cf. glaucum small* |
| DINOFLAGELLATES | *Micranthodinium* | *Kofoidinium sp.* |
| *Undet. Dino.* 20µm | *Oxytoxum sp.* | *"Mandrake Root"* |
| *Undet Cyst* | *Oxytoxum scolopax* | *Noctiluca zoospore* |
| *Brachydinium sp.* | *Prorcentrum sp* 15µm | *Ornithocercus heteroporus* |
| *Ceratium arietinum* | *Prorcentrum sp* 20µm | *Ornithocercus quadratus* |
| *Ceratium azoricum* | *Prorocentrum balticum* | *Oxytoxum sp.* |
| *Ceratium boehmii* | *Prorocentrum compressum* | *Oxytoxum lativelatum* |
| *Ceratium candelabrum* | *Prorocentrum dentatum* | *Oxytoxum milneri* |
| *Ceratium carrense* | *Prorocentrum lenticulatum* | *Oxytoxum nanum* |
| *Ceratium declinatum* | *Prorocentrum gracile* | *Oxytoxum reticulatum* |
| *Ceratium extensum* | *Prorocentrum cf magnum* | *Parahistoneis sp.* |
| *Ceratium furca* | *Prorocentrum micans* | *Podolampas bipes* |
| *Ceratium fusus* | *Prorocentrum cf. minimum* | *Podolampas palmipes* |
| *Ceratium geniculatum* | *Prorocentrum rostratum* | *Protoperidinium sp.* |
| *Ceratium gibberum* | *Prorocentrum triestinum* | *Protoperidinium bipes* |
| *Ceratium hexacanthum* | *Prorocentrum vaginula* | *Protoperidinium brevipes* |
| *Ceratium horridum* | *Pyrocistis lunula* | *Protoperidinium brochii* |
| *Ceratium limulus* | *Pyrocistis fusiformis* | *Protoperidinium curtipes* |
| *Ceratium lineatum* | *Pyrocistis noctiluca* | *Protoperidinium depressum* |
| *Ceratium macrocerus* | *Scripsiella sp.* | *Protoperidinium globosum* |
| *Ceratium "minutum"* | *Amphidoma caudata* | *Protoperidinium oceanicum* |
| *Ceratium pentagonum* | *Amphisolenia globosa* | *Protoperidinium ovum* |
| *Ceratium paradoxoides* | *Blepharocystis sp* | *Protoperidinium pallidum* |
| *Protoperidinium cf. pyriforme Ceratium platycorne* | *Ceratocorys armata* |  |
| *Protoperidinium saltans* | *Syracosphaera pirus* |  |
| *Protoperidinium cf. steinii* | *Holococcolithophorid* 10µm |  |
| *Ptychodiscus noctiluca* | *Holococcolithophorid* 5µm |  |
| *Ptychodiscus noctiluca Small* | *Syracosphaera sp.* 10µm |  |
| *Pyrophacus horologicum* | *Syracosphaera sp.* 15µm |  |
| *Undet. peridinian Small* | *Syracosphaera sp.* 20µm |  |
| *Undet. peridinian Large* | *Syracosphaera pulchra* |  |
| *Undet. Gymnodinium Large* | *Thoracosphaera heimii* |  |
| *Torodinium sp.* | *Emiliania huxleyi* |  |
| *Torodinium sp. Small* | *Gephyrocapsa sp.* |  |
| *Warnowia* | *Gephyrocapsa oceanica* |  |
|  | *Helladosphaera sp.* |  |
| FLAGELLATES | *Thorosphaera flabellata* |  |
| *Cryptomonad* | *Umbillicosphaera hulburtiana* |  |
| *Euglenoid* | *Umbillicosphaera sibogae* |  |
| *Pyramimonas sp* | *Umbellosphaera sp.* |  |
| *Colonial flagellate* 3µm |  |  |
| *Flagellate* 15µm |  |  |
| *Phaeocystis sp.* |  |  |
| *"Oolithus"* |  |  |
| *Alisphaera sp.* |  |  |
| *Acanthoica quattrospina* |  |  |
| *Anoplosolenia braziliensis* |  |  |
| *Anthosphaera sp.* |  |  |
| *Braarudosphaera bigelowii* |  |  |
| *Calcidiscus leptoporus* |  |  |
| *Calciosolenia murrayi* |  |  |
| *Calciopappus sp.* |  |  |
| *Coccolithus pelagicus* |  |  |
| *Calyptrosphaera sp.* |  |  |
| *Calyptrosphaera sp. A* |  |  |
| *Calyptrosphaera sp. B* |  |  |
| *Crenalithus sessilis* |  |  |
| *Crystallolithus cf rigidus* |  |  |
| *Caneosphaera molischii* |  |  |
| *Discophaera tubifer* |  |  |
| *Florisphaera profunda* |  |  |
| *Hallopappus sp.* |  |  |
| *Helicosphaera carteri* |  |  |
| *Deutschlandia anthos* |  |  |
| *Helicosphaera hyalina* |  |  |
| *Michaelsarsia elegans* |  |  |
| *Ophiaster sp.* |  |  |
| *Pontosphaeracf. syracusana* |  |  |
| *Rhabdosphaera claviger* |  |  |
| *Scyphospaera apsteinii* |  |  |
|  |  |  |
