# Supplementary material for: Phytoplankton Biogeography and Community Stability in the Ocean
Source: PLoS One. 2010 Apr 2;5(4):e10037. doi: 10.1371/journal.pone.0010037 (PMC2848864; doi:10.1371/journal.pone.0010037)
Supplement: Table S3 — Geographic coordinates, sample depth and age for diatom fossil records. (0.11 MB DOC) [file pone.0010037.s007.doc]

| M12392 | | M16772-2 | | GeoB1710 | |
| --- | --- | --- | --- | --- | --- |
| Depth (cm) | Age (ky) | Depth (cm) | Age (ky) | Depth (cm) | Age (ky) |
| 3 | 3.0 | 3.8 | 1.09 | 1.5 | 0.4 |
| 10 | 4.7 | 9.8 | 2.83 | 10.5 | 2.7 |
| 20 | 6.7 | 15.8 | 4.57 | 21.5 | 5.6 |
| 28 | 8.7 | 21.8 | 6.32 | 31.5 | 7.5 |
| 60 | 11.0 | 33.8 | 9.8 | 41.5 | 9.2 |
| 70 | 12.0 | 38.5 | 17.1 | 52 | 11 |
| 80 | 13.0 | 47.5 | 18.3 | 62.5 | 12.8 |
| 90 | 13.5 | 67.5 | 22.26 | 73.5 | 14.7 |
| 100 | 14.5 | 87.5 | 26.22 | 83.5 | 16.4 |
| 110 | 15.3 | 107.5 | 30.31 | 93 | 18 |
| 120 | 15.9 | 126.5 | 35.09 | 113.5 | 20.9 |
| 130 | 16.6 | 147.5 | 39.87 | 123.5 | 22.4 |
| 140 | 17.3 | 167.5 | 44.65 | 133.5 | 23.8 |
| 150 | 18.0 | 187.5 | 49.43 | 143.5 | 25.2 |
| 160 | 18.7 | 207.5 | 54.21 | 153.5 | 26.6 |
| 170 | 19.0 | 227.5 | 58.47 | 173.5 | 29.8 |
| 180 | 20.0 | 247.5 | 64.5 | 193.5 | 33.1 |
| 190 | 20.7 | 267.5 | 66.7 | 213.5 | 36.4 |
| 200 | 21.4 | 287.5 | 68.9 | 232.5 | 39.6 |
| 210 | 22.1 | 307.5 | 71.1 | 252.5 | 42.9 |
| 220 | 22.8 | 327.5 | 79.5 | 272.5 | 46.2 |
| 230 | 23.5 | 347.5 | 86.83 | 292.5 | 49.6 |
| 240 | 24.1 | 367.5 | 91.93 | 302.5 | 51.2 |
| 250 | 24.8 | 387.5 | 94.77 | 312.5 | 52.9 |
| 275 | 26.2 | 407.5 | 99.85 | 322.5 | 54.9 |
| 300 | 29.9 | 427.5 | 105.93 | 352.5 | 61.3 |
| 325 | 32.5 | 447.5 | 110.8 | 372.5 | 65.9 |
| 350 | 36.4 | 467.5 | 114.14 | 382.5 | 67.9 |
| 375 | 39.3 | 487.5 | 117.49 | 402.5 | 71.9 |
| 400 | 41.9 | 507.5 | 120.83 | 422.5 | 75.9 |
| 425 | 45.8 | 527.5 | 123.75 | 442.5 | 79.9 |
| 450 | 48.4 | 547.5 | 127.06 | 462.5 | 84.6 |
| 475 | 52.3 | 567.5 | 133.01 | 482.5 | 89.5 |
| 500 | 54.9 | 587.5 | 135.3 | 502.5 | 94.9 |
| 520 | 57.6 | 607.5 | 138.65 | 522.5 | 100.2 |
| 540 | 60.6 | 627.5 | 142 | 542.5 | 105.5 |
| 560 | 63.6 | 647.5 | 144.43 | 583.5 | 115.9 |
| 580 | 66.6 | 667.5 | 146.87 | 603.5 | 120.9 |
| 600 | 69.6 | 687.5 | 149.3 | 622.5 | 125.5 |
| 620 | 77.1 | 707.5 | 154.5 | 642.5 | 130.2 |
| 660 | 88.9 | 727.5 | 162.8 | 652.5 | 132.6 |
| 700 | 100.4 |  |  | 662.5 | 134.9 |
| 740 | 108.4 |  |  | 672.5 | 136.7 |
| 800 | 118.6 |  |  | 693 | 140.5 |
| 840 | 125.1 |  |  | 702.5 | 142.2 |
| 844 | 126.2 |  |  | 712.5 | 144.1 |
|  |  |  |  | 722.5 | 145.9 |
|  |  |  |  | 732.5 | 147.4 |
|  |  |  |  | 742.5 | 148.8 |
|  |  |  |  | 752.5 | 150.2 |
|  |  |  |  | 762.5 | 152.1 |
|  |  |  |  | 782.5 | 157.1 |
|  |  |  |  | 802.5 | 162.1 |
|  |  |  |  | 822.5 | 167.1 |
|  |  |  |  | 842.5 | 172.4 |
|  |  |  |  | 863.5 | 178.6 |
|  |  |  |  | 882.5 | 183.9 |
|  |  |  |  | 902.5 | 189 |
|  |  |  |  | 922.5 | 195.6 |
|  |  |  |  | 942.5 | 203 |
|  |  |  |  | 962.5 | 211.4 |
|  |  |  |  | 982.5 | 219.3 |
|  |  |  |  | 1002.5 | 226.1 |
|  |  |  |  | 1013.5 | 229.6 |
|  |  |  |  | 1032.5 | 235 |
|  |  |  |  | 1052.5 | 242.4 |

Table S3. Geographic coordinates, sample depth and age for diatom fossil records.
