# Supplementary material for: Phytoplankton Biogeography and Community Stability in the Ocean
Source: PLoS One. 2010 Apr 2;5(4):e10037. doi: 10.1371/journal.pone.0010037 (PMC2848864; doi:10.1371/journal.pone.0010037)
Supplement: Table S4 — Diatom species list in sedimentary records. (0.06 MB DOC) [file pone.0010037.s008.doc]

Table S4. Diatom species list in sedimentary records.

CENTRALES

*Actinocyclus curvatulus* Janisch

*eherenbergii* Ralfs

*ellipticus*  Grunow

*normanii* (Greg.) Husted

*octonarius* Ehrenberg

*subtilis* (Greg.) Ralfs

*Actinoptychus adriaticus* Grunow

*senarius* (Ehr.) Ehrenberg

*splendens* (Shadb.) Ralfs

*Anaulus birostratus* Grunow

*mediterraneus* var *intermedia* Grunow

*Asterolampra marylandica* Ehrenberg

*Asteromphalus arachne* (Bréb.) Ralfs in Pritchard

*elegans* Greville

*flabellatus* (Bréb.) Greville

*heptachis* (Bréb.) Ralfs

*hookeri Ehrenberg*

cf. *robustus* Castracane

*sarcophagus* Wallich

*Aulacodiscus kittonii* Arnott

*Aulacosira granulata* (Thwaites) Crawford

*islandica* (O. Muller) Crawford

*Auliscus sculptus* (W.Sm.) Ralfs

*Azpeitia africana* (Janisch ex A. Schmidt)

*crenuloides* P.A. Sims

*neocrenulata* (Van Landingham) Fryxell & Watkins

*nodulifera* (Sch.) Fryxell & Sims

*tabularis* (Grun.) Fryxell & Sims

*tabularis* var. *egregius* (Rattray) Fryxell & Sims

*Bacteriastrum hyalinum* Lauder

*Biddulphia alternans* (Bail.) Van Heurck

cf. *laevis* Ehrenberg

*pulchella* Gray

*regina* W. Smith

*tuomeyi* (Bail.) Roper

*Cerataulus smithii* Ralfs

*turgidus* (Ehr.) Ehrenberg

*Chaetoceros diadema* (Ehr.) Gran

*gracilis* Schutt

*lorenzianum* Grunow

*radicans* Gran

*teres* Cleve

unidentified vegetative cells

unidentified spores

*Coscinodiscus apiculatus* Ehrenberg

*argus*  Ehrenberg

*asteromphalus* Ehrenberg

*curvatulus* Grunow

*curvatulus var. minor* (Ehr.) Grunow

*granii* Gough

cf. *Hauckii* Grunow

*marginatus* Ehrenberg

*oculus-iridis* Ehrenberg

*perforatus* var. *Pavillardi* (Forti) Hustedt

*radiatus* Ehrenberg

*rothii* (Ehr.) Grunow

*Cyclotella caspia Grunow*

*comensis* Grunow

*distinguenda* Hustedt

cf**.** *kutzingiana* Thwaites

*litoralis* Lange & Syvertsen

*meneghiniana* Kutzing

*ocellata* Pantocsek

*operculata* (Ag.) Kutzing

*striata* (Kutz.) Grunow

*Eucampia antartica* Castracana

*Ethmodiscus rex* (Wall.) Hendey

*Groentvedia elliptica* Hendey

*Hemialus ambiguus* Grunow

cf. *hauckii* Grunow

*Hemidiscus cuneiformis* Wallich

*Hyalodiscus cf. laevis* Ehrenberg

cf. *radiatus* (O'Meara) Grunow

*Leptocylindrus spp.* Cleve (spores)

*danicus* Cleve (spores)

*Lithodesmium undulatum* Ehreberg

*Melosira*  *ambigua* (Grun.) O.Muller

*arenaria* Moore

*distans* (Ehr.) Kutzing

*dubia* Kutzing

*italica* (Ehr.) Kutzing

*roeseana* Rabhenhoest

*varians* Agardh

*westii* Smith

*Odontella aurita* (Lyngbye) Agardh

*longicruris* (Greville) Hoban

*mobiliensis* (Bail.) Grunow & Van Heurk

*regia* (M. Schutze) Ostenfeld

*Paralia sulcata* (Ehr.) Cleve

*Planktoniella sol* (Wall) Schutt

*Podosira maxima* (Kutz.) Grunow

*stelliger* (Bailey) Mann

*Psammodiscus nitidus* (Gregory) Round

*Pseudotriceratium*

*Rhizosolenia alata* Brightwell

*bergonii* H. Pergallo

cf**.** *calcar-avis* M. Schultze

*clevei* Ostenfeld var. *clevei*

*debyana H. Peragallo*

*hebetata* Bailey

*hebetata* f. *semispina* (Hensen) Gran

cf. *setigera* Brightwell

*styliformis*  Brightwell

*Roperia tesselata*  (Roper) Grunow ex Van Heurck

*Rossiella paleacea* (Grun.) Desikachary and Maheshwari

*Skeletonema* cf *costatum* (Greville) Cleve

*Stephanodiscus astrea* (Ehr.) Grunow

*dubius*  Grunow = Matrensis Pantocsek

cf. *hantzschii*  Grunow

*minutus* Grunow = Matrensis Pantocsek

*Stephanopyxis cf. broschii* Grunow

*turris*  (Grev.) Ralfs

*Stictodiscus parallelus var. balearica* Grunow

*Stellarina stellaris* (Roper) Hasle & Sims

*Thalassiosira aestivalis* Gran

*anguste-lineata*  (A. Schmi.) Fryxell & Hasle

*binata* Fryxell

*conferta* Hasle

*decipiens* (Grunow ex Van Heurck) Jorgensen

*delicatula* (Ostenfeld) Hasle

*diporocyclus* Hasle

*eccentrica*  (Ehr.) Cleve

cf**.** *eccentrica* (minima)

*ferelineata* Hasle & G. Fryxell

*gracilis* (Karsten) Hustedt var*. gracilis*

*hendeyi* Hasle & G. Fryxell

*leptopus*  (Grun.) Hasle & Fryxell

*lineata* Jouse

*lineoides* Herzig & G. Fryxell

*mendiolana* Hasle & Heimdal

*minuscula* Krasske

*monoporocyclus* Hasle

*oceanica* Hasle

*oestrupii*  (Ostenf.) Hasle

*oestrupii* var. *venrickae* G. Fryxell & Hasle

*pacifica* Gran & Angst

*poro-irregulata* Hasle & Heimdal

*poroseriata* (Ramsfjell) Hasle

*subtilis* (Ostenfeld) Gran

*symmetrica* Fryxell & Hasle

*tumida* (Janisch) Hasle

sp3 (similar to C. radiatus)

*Triceratum antediluvianum*  (Ehr.) Grunow

*dubium* Brightwell

*favus* Ehrenberg

*formosum f. quadrangularis* Brightwell

*repletum* Greville

*Xanthiopyxis* Ehrenberg

sp1 (bump on side)

sp2 (bump on center)

PENNALES

*Achnanthes biasolettiana* (Kutz.) Grunow

*brevipes* Agardh

*clevei* Grunow

cf. *danica* (Flogel) Grunow

*dispar* Cleve

*lillyeborgei* Grunow

*longipes* Agardh

cf*. microcephata* (Kutz.) Cleve

*saxonica* Krasske

*Amphicampa hemicyclus* (Ehr.) Karst

*Amphora grevilleana* Gregory

*groeffi* var. *minor* Pergallo

*hyalina* Kutzing

*ostrearia* de Brebisson ex Kutzing var. *ostrearia*

*spectabilis* Gregory

*Campiloneis grevillei* (Wm. Smith) Grunow

*Cocconeis costata* Gregory

*debeyi* Hustedt

cf *diminuta* Pantocsek

*disculus* (Schumann) Cleve

*disrupta* Gregory

*distans* Gregory

cf. *grata* A. Schmith

*fulminensis* (Grun.) Peragallo

*hustedtii* Krasske

*ornata* Gregory

*pinnatta* Gregory

*placentula* Ehrenberg

*pseudomarginata* Gregory

*quanerensis* (Grunow) Schimdt

*scutellum* Ehrenberg var. *scutellum*

*scutellum* var. *parva* Grunow ex Cleve

*thumensis* A. Mayer

*Cymatopleura elliptica* (Bréb.) W. Smith

*Delphineis* karstenii

*kippae* Sancetta

*surirella*  (Ehr.) W. Andrews

# *surirelloides* (Simonsen) G. W. Andrews

*Denticulopsis hustedtii* (Simonsen & Kanaya) Simosen

*Diatoma vulgare* Bory

*Dimeregramma dubium* Grunow

*fulvum* (Greg.) Ralfs

*marinum* (Greg.) Ralfs

*minor* (Greg.) Ralfs

*Diploneis bombus*  Ehrenberg

*chersonensis* Grunow

*coffaeiformis* (A.S.) Cleve

*constricta* (Grunow) Cleve

*crabro* Ehrenberg

*cynthia* (A.S.) Cleve

*dalmatica* (Grun.) Cleve

cf. *florinae* (A.S.) Cleve

*fusca* (Greg.) Cleve

*incurvata* (Greg.) Cleve

*incurvata* var. *dubia* Hustedt

*lineata* (Donk.) Cleve

*mediterranea* (Grun.) Cleve

*minuta* Petersen

cf*. oculata* (Breb.) Cleve

cf. *papula* (A.S.) Cleve

cf. *parma* Cleve

*smithii*  Brebisson

*subcinta* (A.S.) Cleve

*suborbicularis* (Greg.) Cleve

*subovalis* Cleve

cf. *vetula* (A.S.) Cleve

*Epithemia argus* Kutzing

*sorex* Kutzing

*zebra* (Cleve-Euler) var. longicornis

*Eunotia papilio* (Grun.) Hustedt

*pectinalis* (Dillw.) Rabenhorst

*polydentula* Brun

cf *praerupta* var. *muscicola* Petersen

*Fragillaria bicapitata* A. Mayer

*brevestriatra* Grunow

*capucina* Desmazieres

*construens* (Ehr.) Grunow

*inflata* (Heid.) Hustedt

*lapponica* Grunow

*lapponica* Grunow

*leptostauron* (Ehr.) Hustedt

*leptostauron* var. *dubia* Grunow

*pinnata* Ehrenberg

*virescens* Ralfs

*Fragilariopsis doliolus* (Wallich) Medin & Sims

*kerguelensis* (O'Meara) Hustedt

*Gomphonema* cf *augur* Cleve

cf. *gracile* Cleve

*Grammatophora angulosa* Ehrenberg

*hamulifera* Kutzing

*macilenta* Wm. Smith

*marina* (Lyngbye) Kutzing

*oceanica* (Ehr.) Grunow

*serpentina* (Ralfs) Ehrenberg

*Hantzschia amphioxys* (Ehr.) Grunow

*virgata* (Roper) Grunow var. *virgata*

*Lioloma elongatum* (Grunow) Hasle

*pacificum* (Cupp) Hasle

*Navicula arenaria* Donkin

*atlantica* (Sch.) Cleve

*clavata* Gregory

cf. *cruciculoides* Brockmann

cf. *directa* (Wm. Smith) Ralfs

*distans* (Wm. Smith) Schmidt

*elegans* Wm. Smith

*ergadensis* var. *minor*  (Peragallo) Hendey

*expansa* Hagelstein

*galikii* Pant. Cleve

*hennedyii* Wm. Smith

*hennedyoides* Hustedt

*hochstetteri* Grunow

cf. *humerosa* De Brebisson

*laevistriatae* group. Cleve

*lyratae* gr. Cleve

*marina*  Ralfs in Pritchard

*palpebralis* de Brebisson ex Wm. Smith

*praetexta* Ehrenberg

*punctatae* group Cleve

*Nitzschia angularis* Wm. Smith

*bicapitata*  Cleve

*bifurcata* Kaczmarska & Licea

*braadurii* Hasle

*elegans* (Lagst.) A. Cleve

*fraudulenta* Cleve

*kerguelensis* (O'Meara) Hasle

*littoralis* Grunow

*marina*  Grunow

*navicularis* (de Brebisson ex Kutzing) Grunow

*panduriformis* Gregory

*punctata* (Wm. Smith) Grunow var. *punctata*

*pungens* Cleve

*recta* Hantzsch. Grunow

*socialis* Gregory var. *socialis*

*subfraudulenta* Hasle

*subpacifica* Hasle

*thermalis* Kutz. Grunow

*Opephora gemmata* (Grun.) Hustedt

*marina* Gregory

*martyi* Heribaud

*pacifica* (Grunow) Petit

*schwartzii* (Grunow) Petit

*Pseudonitzschia australis* Frenguelli

*fraudulenta* (Cleve) Hasle

*seriata* (Cleve) H. Peragallo

*Pinnularia* *ambigua* Cleve

cf. *rectangulata* (Greg.) Rabenhorst

*Plagiogramma interruptum* Gregory

*pulchellum* Greville

*staurophorum* (Greg.) Heiberg

*Pleurosigma*  cf. *angulatum* (Quekett) Wm. Smith

cf. *cuspidatum* Cleve

*strigosum* Wm. Smith

*Pseudoeunotia doliolus*  Wallich

*Rhaphoneis amphiceros*  Ehrenberg

*Rhabdonema arcuatum* (Lyngbye) Kutzing

cf. *torelli* Cleve

*Rhopalodia gibba* (Ehr.) O. Muller var. *gibba*

*gibberula (*Ehr.) O.Muller

*musculus* (Kz.) A.Cleve

*Sceptroneis caducea* Ehrenberg

*Stauroneis acuta* W. Smith

*amphioxis* Gregory var. *amphioxys*

*Surirella amoricana*  Peragallo

*fastuosa* (Ehr.) Kutzing

*intermedia* Cleve-Euler

*ovalis* de Brebisson

*Synedra gaillonii* (Bory) Ehrenberg

cf. *hennedyana* Gregory

*tabulata* (Ag.) Kutzing

*ulna* (Nitzsch.) Ehrenberg

*Tabellaria fenestrata* (Lyngb.) Kutzing

*Thalassionema bacillare* (Heiden in Heiden & Kolbe)

*nitzschioides*  (Grunow) Mereschkowsky

*nitzschioides* (Grunow) Hustedt var. *nitzschioides*

*nitzschioides var. parva*  Heiden & Kolbe

*Thalassiothrix cf longissima*  Cleve & Grunow

*Trachyneis aspera* (Ehr.) Cleve
